# Necrotizing pneumonia and purulent meningitis caused by bloodstream infection of CA-MRSA in a child: A rare case report

**DOI:** 10.3389/fped.2022.1045774

**Published:** 2022-12-08

**Authors:** Peng Li, Lizhong Dai, Ke Yuan, Chunlan Song, Yibing Cheng, Yu Jing, Na Chen, Junhao Cui, Yangji Wang, Shuqin Fu

**Affiliations:** ^1^ Department of Emergency Medicine, Children's Hospital Affiliated of Zhengzhou University, Henan Children's Hospital Zhengzhou Children's Hospital, Zhengzhou, China; ^2^ National Joint Engineering Research Center for Infection Diseases and Cancer Diagnosis, Changsha, China

**Keywords:** neurology, respiratory medicine, infectious diseases, community-acquired methicillin-resistant *Staphylococcus aureus*, necrotizing pneumonitis, suppurative meningitis, young children

## Abstract

**Case presentation:**

We report the case of a girl aged 2 years and 10 months who had fever for 2 days, vomiting, poor mental status for 1 day, and one episode of convulsions.

**Symptoms and signs:**

The patient experienced a rapid onset of symptoms with fever, vomiting, and convulsions. Upon physical examination on admission, she presented with the following: temperature 38.6°C; pulse 185 beats/min; respiration 49 beats/min; blood pressure 89/51 mmHg; drowsiness; piebald skin all over her body; rice-grain-sized pustular rashes scattered on the front chest and both lower limbs, protruding from the surface of the skin; bilateral pupils that were equal in size and a circle with a diameter of about 3.0 mm, and slow light reflex; cyanotic lips; shortness of breath; positive for the three-concave sign; a small amount of phlegm that could be heard in both lungs; capillary refill time of 5 s; cold extremities; and a positive Babinski sign.

**Diagnostic method:**

A chest computed tomography scan showed multiple nodular and flake-like high-density shadows of varying sizes in each lobe in bilateral lungs, and a cavity with blurred edges could be seen in some nodules. A cranial magnetic resonance imaging examination demonstrated that the hyperintensity of diffusion-weighted imaging could be observed on the left cerebellar hemisphere and left parietal blade. Blood cultures, sputum, cerebrospinal fluid, and bronchoalveolar lavage fluid (BALF) by fiberoptic bronchoscopy all indicated the growth of methicillin-resistant *Staphylococcus aureus* (MRSA).

**Treatment methods:**

After admission, the child was given meropenem combined with vancomycin, cefoperazone sulbactam combined with rifamycin, linezolid (oral) for anti-infection successively, and other adjuvant therapies.

**Clinical outcomes:**

The patient recovered clinically and was discharged from our hospital.

**Recommended readers:**

Neurology; Respiratory Medicine; Infectious Diseases Department.

## Introduction

The definition of community-acquired methicillin-resistant *Staphylococcus aureus* (CA-MRSA) is 1. a bacteria found in an outpatient or inpatient within 48 h of isolation of a MRSA strain in a patient with pneumonia 2. no history of hospitalization or contact with medical devices within 1 year (1). At present, the prevalence of CA-MRSA in China is still unclear, and there is a lack of relevant literature about CA-MRSA bloodstream infection leading to pneumonia complicated with encephalitis in children, an area that requires more clinical research (2). This study analyzed and discussed the clinical diagnosis and treatment process of a child with purulent meningitis and necrotizing pneumonia caused by a CA-MRSA bloodstream infection, so as to improve the understanding of the disease and provide a reference for rational drug use in clinical practice.

## Clinical information

### General information

A girl aged 2 years and 10 months was admitted to our hospital on 11 February 2019, due to fever for 2 days, vomiting, poor mental status for 1 day, and one episode of convulsions. Two days before admission, the child had a fever without an obvious cause, and the body temperature was in the range of from 37.3°C to 39.8°C. One day before admission, the patient appeared to have poor mental status, accompanied by non-projectile vomiting approximately 7–8 times. The vomitus was yellowish-brown in color, in varying amounts, accompanied by abdominal discomfort; the specific location and nature were unknown. During this period, rice-grain-sized pustular rashes scattered on her chest and both lower limbs, protruding from the surface of the skin, without itching. Ceftazidime, oseltamivir, traditional Chinese medicine, and calamine lotion were given in the local hospital, and the therapeutic effect was poor. The child went into convulsions 7 h, which manifested as a pale face, upturned eyes, shaking limbs, clenched hands, no cyanosis of the lips, closed teeth, urine and stool incontinence, lasting for approximately 5 min, and was relieved after symptomatic treatment in the local hospital. The patient was then admitted to our hospital for further diagnosis and treatment.

Upon admission, the results of her physical examination were as follows: body temperature 38.6°C; pulse 185 beats/min; respiration 49 beats/min; blood pressure 89/51 mmHg; and transcutaneous oxygen saturation 89%. The patient was drowsy, with piebald skin all over her body and rice-grain-sized pustular rashes scattered on her chest and both lower limbs, protruding from the surface of the skin. Her bilateral pupils were equal in size and round (straight 3.0 mm), with a sluggish light reflex. Her lips were cyanotic, and she experienced a shortness of breath. She was positive for the three-concave sign and had thick breath sounds in both lungs, with a small amount of phlegm heard in both lungs. Her heart sounds were strong and rhythmic, and no murmur was heard in each valve auscultation area. The abdomen was soft, and the liver was 3 cm below the lower edge of the ribs. The spleen was not palpable, and the bowel sounds were normal. The capillary refill time was 5 s, the ends of her extremities were cold, and the Babinski sign was positive.

The patient had previously been healthy. The parents of the child were healthy and non-consanguineous. They denied a history of infectious diseases such as hepatitis, tuberculosis, and the history of close contact with infectious patients; they also denied any family history of genetic and metabolic diseases. The child had been vaccinated once with free vaccines stipulated by the state, but not with PCV-13 at their own expense.

### Inspection

Initial blood investigations revealed a white blood cell count (WBC) of 4.21 × 10^9^/L with 72.6% neutrophils and normal hemoglobin and platelets. The arterial *Mycoplasma pneumoniae* serological test was negative. Levels of serum C-reactive protein (CRP) and procalcitonin (PCT) were both markedly elevated at 108 mg/L and 15.2 ng/ml, respectively. The D-dimer level was 17.93 μg/ml.

The cerebrospinal fluid (CSF) examination revealed yellowish and slightly turbid features: WBC 0.05 × 10^9^/L with 82% mononuclear cells and 18.0% polynuclear cells; and CSF protein 1020.8 mg/L. Pandy's reaction showed slight white turbidity (+), glucose 1.7 mmol/L, and chlorine 111 mmol/L.

The chest computed tomography (CT) scan showed multiple nodular and flake-like high-density shadows of varying sizes in each lobe in bilateral lungs, and a cavity with blurred edges could be seen in some nodules. The dorsal segments on both sides were obviously thickened.

A cranial magnetic resonance imaging (MRI) examination demonstrated that the hyperintensity of diffusion-weighted imaging (DWI) could be observed on the left cerebellar hemisphere and left parietal blade. Blood cultures, sputum, CSF, and bronchoalveolar lavage fluid (BALF) by fiberoptic bronchoscopy all indicated the growth of MRSA.

A brain MRI scan revealed a flake DWI high signal in the left cerebellar hemisphere and left parietal lobe, suggesting cytotoxic edema.

Samples from blood, sputum, CSF, and BALF were sent to the laboratory for inoculation in a medium for bacterial culture. The Phoenix TM-100 automated bacterial identification and drug sensitivity system was used to identify and analyze the bacteria. The results were judged according to the 2019 standards of the Clinical and Laboratory Standards Institute (CLSI). The reports indicated the growth of MRSA, and the drug sensitivity results indicated that it was sensitive to vancomycin, compound sulfamethoxazole, linezolid, and rifampicin.

First, the samples were centrifuged at 4,000 rpm for 10 min at 4°C within 8 h of collection. Then, samples were transferred to new sterile tubes. Next, the manufacturer's standard operational procedures were used to extract DNA from 300 μl of samples using nucleic acid extraction or a purification reagent from Sansure Biotech (XCXB No. 20150021). The evaluation reagent was the Six Respiratory Pathogens Nucleic Acid Diagnostic Kit from Sansure Biotech (PCR fluorescence probe method), and the detection instrument was a ABI 7500 real-time fluorescent PCR (Thermo Fisher). The results of lymphocyte subsets test displayed the following: T lymphocytes (CD3+) 43%; T8 lymphocytes (CD3 + CD8+) 13%; T4 lymphocytes (CD3 + CD4+) 28%; CD4/CD8 2.09; NK cells (CD16 + 56+) 11%; and B cells (CD19+) 45%. Immune function showed the following: immunoglobulin M 0.74 g/L; immunoglobulin A 0.75 g/L; immunoglobulin G 5.28 g/L; complement C3 0.84 g/L; and complement C4 0.14 g/L.

### Diagnosis

#### Purulent meningitis (CA-MRSA)

The patient had clinical manifestations such as fever, vomiting, poor mental status, and convulsions. A physical examination showed that the patient had a disturbance of consciousness, a dullness of bilateral pupillary light reflex, and a positive bilateral Babinski sign. An auxiliary examination results showed that CRP and PCT increased significantly.

The CSF examination revealed yellowish and slightly turbid features: WBC 0.05 × 10^9^/L with 82% mononuclear cells, chlorine 111 mmol/L, glucose 1.7 mmol/L, which was significantly decreased, and CSF protein 1020.8 mg/L, which was significantly increased. The CSF smear and culture showed MRSA.

#### Necrotizing pneumonia (CA-MRSA)

The patient had clinical manifestations including fever, vomiting, poor mental status, and convulsions. A physical examination revealed that the patient had cyanotic lips, a shortness of breath, a positive three-concave sign, and sputum sounds in both lungs. An auxiliary examination showed that levels of CRP and PCT were significantly increased. The lung CT scan showed that multiple nodular and scaly increased density shadows of different sizes were seen in each lobe of the two lungs, and some nodules with blurred edges of cavities could be seen. Sputum and BALF cultures demonstrated MRSA.

#### Skin abscess (CA-MRSA)

The patient had clinical manifestations such as a fever and rash. A physical examination revealed rice-grain-sized pustular rashes scattered on the chest and both lower extremities, protruding from the surface of the skin. An auxiliary examination showed that serum CRP and PCT increased remarkably. A blood smear and culture displayed MRSA.

### Treatment

After the child was admitted to the hospital, meropenem combined with vancomycin was given to fight infection from 11 February to 24 February. The child's rash gradually decreased and scabbed. On the 7th day after admission, the number of white blood cells and protein in the CSF were significantly decreased; however, the recovery of the child's body temperature, fluctuating between 38.5°C and 39.0°C, was unsatisfactory. The use of special grade antibiotics for 14 consecutive days would easily lead to a decrease of the body’s sensitivity to drugs, a decrease in the effects of the drug treatment, and the emergence of other drug-resistant bacteria; therefore, we adjusted the use of antibiotics. From 24 February to 12 March, the antibiotic was replaced with cefoperazone-sulbactam and rifamycin. The body temperature gradually decreased and was normal, and the rash subsided. On the 15th day after admission, the routine and biochemical re-examination results of the CSF were normal, and the lung CT scan was better than before. The child had been hospitalized for a while and her condition had improved. In combination with the drug sensitivity results, the intravenous medication was discontinued and replaced with oral medication. At the same time, the infection control indicators were closely monitored to prepare for the child's clinical recovery and discharge from the hospital. From 12 March to 20 March, the drug was changed to oral linezolid. On the 30th day after admission, the routine and biochemical indexes of the CSF were normal.

The child was also given adjuvant therapy, such as CPAP-assisted ventilation, intracranial decompression to stop convulsions, application of vasoactive drugs to improve circulation, application of electrolytes to maintain internal environment stability, among others.

### Treatment result, follow-up, and outcome

The child was discharged from the hospital on 20 March with clinical recovery. Two blood cultures, two sputum cultures, two CSF cultures, and BALF culture experiments were free of bacterial growth before discharge.

**Figure 1:**
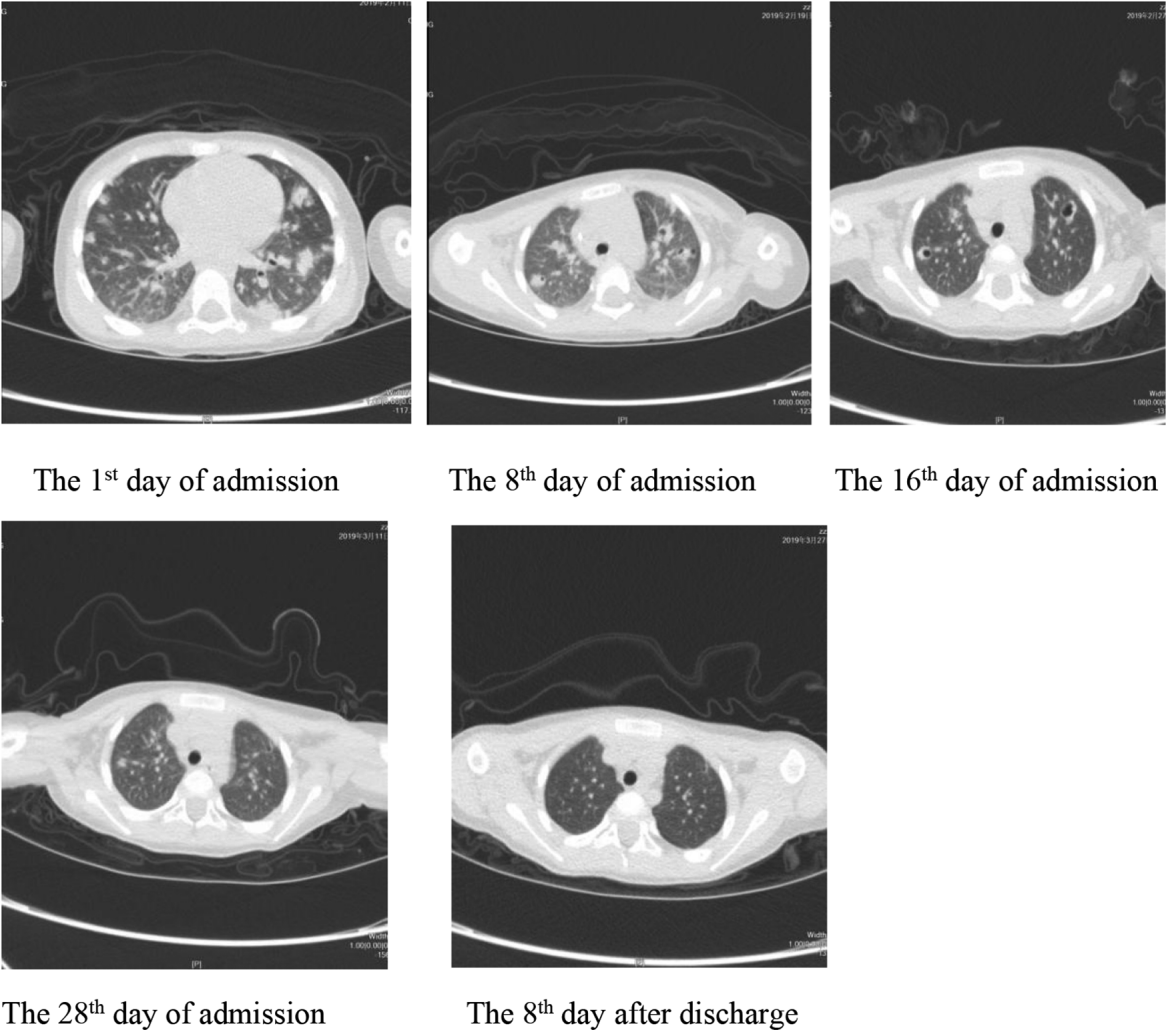
Imaging manifestations, transformation, and outcomes of patients.

On 27 March, the patient's outpatient follow-up and re-examination of the lung CT scan showed that the texture of both lungs was thick, and that there were still patches of increased density shadows in each lobe of both lungs; nodular shadows were seen in the lower lobe of the left lung, and several cavities were still visible in the upper lobe of the left lung and the lower lobe of the right lung. Compared with the previous CT examination, the shadow range was significantly reduced, the number of cavities was significantly decreased, and the amount of pleural effusion on the left side was reduced (Figure 1).

## Discussion

In the past, MRSA was mostly found in hospitalized patients who were immunocompromised, had basic diseases, or who had undergone major surgery. MRSA was a common pathogen of nosocomial infection. In recent years, population mobility has increased, and various antibiotics are widely applied in clinical work. An increasing number of healthy people are also infected with MRSA, resulting in an increase in the prevalence of CA-MRSA year on year (3–5). At present, scholars from various countries have not yet unified the definition of CA-MRSA and our country mainly uses the definition of CA-MRSA from the US Centers for Disease Control (CDC) from 2000 (6). CA-MRSA is usually resistant to β-lactamase antibiotics, and often causes soft tissue and skin infections, which are commonly seen in necrotizing fasciitis and skin abscesses (7). In this study, the patient had impetigo, which is consistent with previous reports of CA-MRSA.

Strains of CA-MRSA are easy to adhere to and destroy respiratory epithelial cells (8), which can cause an uncontrolled inflammatory response, apoptosis of tissue cells, and necrosis of alveolar membranes. These characteristics can cause necrotizing pneumonia, such as lung consolidation, nodules, and cavities, which has severe clinical symptoms, rapid disease progression, and is difficult to treatment (9, 10). The study by Marcinak et al. (11) showed that children infected with CA-MRSA are more likely to have a combination of lower respiratory tract infections. Geng et al. (12) reported that infants and young children are more susceptible to CA-MRSA pneumonia. Zhang et al. (13) also reported a healthy student with MRSA in blood culture. Hardgrib et al. (14) also reported a case of a 13-year-old previously healthy boy with MRSA-induced pneumonia and joint soft tissue infection.

In the present case, the child had a 1-day history of acute onset, with high fever, disturbance of consciousness, and convulsions as the main clinical manifestations. The laboratory examination combined with lung CT scan, head MRI scan, CSF routine, and biochemical and other data, confirmed the diagnosis of suppurative meningitis and necrotizing pneumonia. The child's CSF culture, sputum culture, blood culture, BALF culture, and drug susceptibility test all indicated MRSA infection. Meanwhile, the child had been healthy in the past and had not been hospitalized in the last year; nor had she received medical operations such as surgery, indwelling urinary catheter, or hemodialysis, which conforms to the definition of CA-MRSA by the US CDC. In this case, there were scattered rice-grain-sized pustular rashes on her chest and both lower extremities, and blood culture suggested MRSA. Therefore, it was considered that the child had a skin and soft tissue infection, which led to purulent meningitis and necrotic pneumonia through bloodstream infection of CA-MRSA.

At present, there are only a few reports on necrotizing pneumonia caused by bloodstream infection of CA-MRSA at home and abroad. Specifically, there is a lack of studies on bloodstream infection of CA-MRSA leading to necrotizing pneumonia and suppurative meningitis. In this study, we report a case of bloodstream infection with CA-MRSA leading to both necrotizing pneumonia and septic meningitis in a young child, hoping to draw the attention of pediatric clinicians to CA-MRSA.

## Data Availability

The original contributions presented in the study are included in the article/Supplementary Material, further inquiries can be directed to the corresponding author/s.
